# Alphavirus Identification in Neotropical Bats

**DOI:** 10.3390/v14020269

**Published:** 2022-01-28

**Authors:** Lucía Moreira Marrero, Germán Botto Nuñez, Sandra Frabasile, Adriana Delfraro

**Affiliations:** 1 Sección Virología, Facultad de Ciencias, Universidad de la República, Montevideo 11400, Uruguay; lmoreira@fcien.edu.uy; 2 Programa para la Conservación de los Murciélagos de Uruguay, Museo Nacional de Historia Natural, Montevideo 11000, Uruguay; gbotto@fmed.edu.uy; 3 Departamento de Métodos Cuantitativos, Facultad de Medicina, Universidad de la República, Montevideo 11800, Uruguay; 4 Departamento de Biodiversidad y Genética, Instituto de Investigaciones Biológicas Clemente, Montevideo 11600, Uruguay

**Keywords:** alphavirus, arbovirus, Chiroptera, Uruguay

## Abstract

Alphaviruses (*Togaviridae*) are arthropod-borne viruses responsible for several emerging diseases, maintained in nature through transmission between hematophagous arthropod vectors and susceptible vertebrate hosts. Although bats harbor many species of viruses, their role as reservoir hosts in emergent zoonoses has been verified only in a few cases. With bats being the second most diverse order of mammals, their implication in arbovirus infections needs to be elucidated. Reports on arbovirus infections in bats are scarce, especially in South American indigenous species. In this work, we report the genomic detection and identification of two different alphaviruses in oral swabs from bats captured in Northern Uruguay. Phylogenetic analysis identified Río Negro virus (RNV) in two different species: *Tadarida brasiliensis* (*n* = 6) and *Myotis* spp. (*n* = 1) and eastern equine encephalitis virus (EEEV) in *Myotis* spp. (*n* = 2). Previous studies of our group identified RNV and EEEV in mosquitoes and horse serology, suggesting that they may be circulating in enzootic cycles in our country. Our findings reveal that bats can be infected by these arboviruses and that chiropterans could participate in the viral natural cycle as virus amplifiers or dead-end hosts. Further studies are warranted to elucidate the role of these mammals in the biological cycle of these alphaviruses in Uruguay.

## 1. Introduction

Alphaviruses (*Togaviridae*) are arthropod-borne viruses associated with emerging infectious diseases of public health concern. The genus includes 32 species widely distributed throughout the world. They are single stranded, positive sense RNA, enveloped viruses. Their genome is 11–12 kilobases in size and encodes five structural proteins (C, E3, E2, 6K, and E1) and four non-structural proteins (NSP1, NSP2, NSP3, and NSP4) [1]. Most alphaviruses are mosquito-borne and are pathogenic in their vertebrate hosts and cause a wide variety of diseases in humans, ranging from febrile illness to more severe clinical symptoms, such as arthritis or encephalitis [2].

The intrinsic genetic variability and adaptability of alphaviruses, together with habitat disturbance by human activities and climate changes, are factors that may alter their ecological niche, leading to events of viral emergence or re-emergence. As recent examples, we may cite the emergence of chikungunya (CHIKV) and Mayaro viruses (MAYV) in Central and South America, or the re-emergence of Madariaga virus (MADV) in Central America [2,3,4,5,6].

In South America, alphaviruses from the Venezuelan equine encephalitis virus (VEEV) complex have been responsible for many epidemics and equine epizootics in Venezuela, Colombia, Ecuador, Trinidad, and Peru [7,8]. MADV (eastern equine encephalitis virus complex) has been recently reported in Argentina in mosquito infections but not has yet been associated with human disease. Conversely, Rio Negro virus (RNV), which also belongs to the VEEV complex, was associated only with febrile illness in humans. It was isolated from mosquitoes and rodents, and serological studies indicate the presence of human antibodies in Argentina [9,10]. A recent serologic survey from Paraguay also reported neutralizing antibodies to this virus in the human population [11].

Alphavirus circulation in Uruguay has been detected since 1970 [12]. The presence of EEEV, VEEV, and western equine encephalitis virus (WEEV) was reported in mosquitoes, equines, and humans [12,13,14]. Rio Negro virus is the most geographically widespread species in the country, and its presence was confirmed by molecular detection in mosquitoes and seroprevalence studies in horses. Burgueño et al. (2018) suggest that several alphaviruses may circulate in enzootic cycles in Uruguay, with sporadic epizootic events that involve mosquitoes and vertebrates [14].

Bats (order Chiroptera) are the second most diverse mammal order in the world, after rodents [15]. Regardless of the negative public perception, bats are critical components of terrestrial ecosystems that fulfill critical roles such as arthropod suppression, pollination, seed dispersal, and forest regeneration [15,16,17]. They possess a set of features among mammals, including the potential to fly long distances, migratory and gregarious habits, and longevity, that make them a possible vehicle for zoonotic virus transmission than other animals [18]. In recent years, interest in bats has increased due to them being recognized as harboring many emerging and re-emerging viruses but fewer viruses than those found in rodents [19].

Some of these viruses are pathogenic for humans but do not cause overt pathology in their bat reservoir hosts [20]. However, a recent study found no differences in the number of human-infecting viruses harbored by bats and other orders of mammals and birds, when controlling for species richness [21].

Bats have long been suspected as being a reservoir for arboviruses, including alphaviruses and flaviviruses, but their contribution to arbovirus dynamics is not known and can vary widely. Some flaviviruses such as dengue virus (DENV) and West Nile virus (WNV) have been detected in bats [22,23]. Additionally, serological evidence exists supporting exposure of bats to encephalitic alphaviruses in the field, and experimental data demonstrate the susceptibility of bats to infection with alphaviruses, including VEEV [23]. Recently, molecular and cellular evidence of VEEV has been described in frugivorous bats in Colombia [24].

The transmission of virus from bats to humans or domestic animals requires several factors to be aligned, including the ecological, epidemiological, and behavioral determinants of exposure to pathogens and human factors that may affect the susceptibility of infection [25]. Natural changes in the environment, as well as those produced by human activities, promote new interactions between viruses, vectors, and mammal hosts that can promote virus infections [26,27].

In Uruguay, there are 22 recorded species of bats, belonging to Vespertilionidae, Molossidae, and Phyllostomidae families, comprising frugivorous, insectivore, and hematophagous diets [28]. Although little is known about the viruses they harbor, some studies have been published focusing on the analysis of antigenic and genetic variability of rabies virus [29], spillover events of rabies virus [30,31], and more recently, analyzing the herpesvirus infections and their ecological and conservational significance [32].

The aim of this study was the molecular detection and genetic characterization of alphaviruses in oral swabs collected from bats in Uruguay. We intended to shed light on the viral diversity in bats and, in view of the previous reports on alphavirus circulation in Uruguay, we discuss their potential ecological role in the biological cycles of these health-threatening arboviruses.

## 2. Materials and Methods

### 2.1. Sample Collection

**Figure 1:**
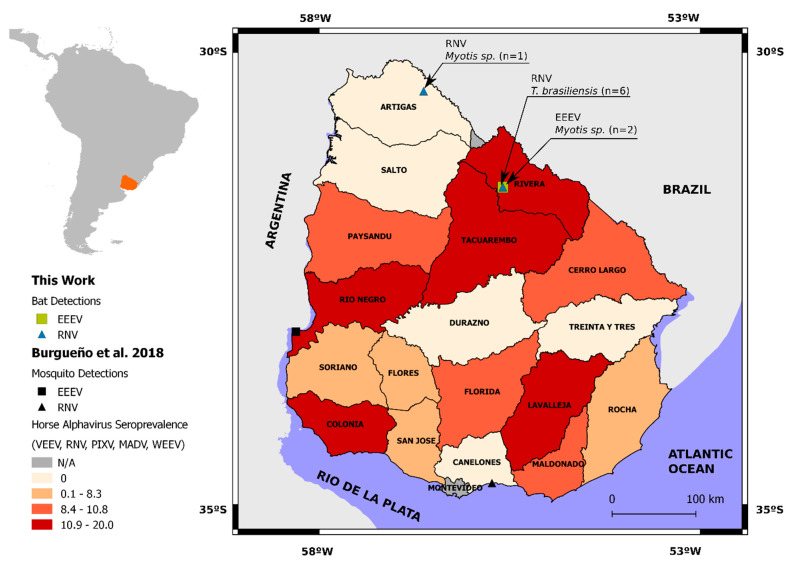
Geographical place of Uruguay in South America and its political division. Alphavirus seroprevalence in horses is depicted in color gradation by percentage ranks; mosquito-positive samples are depicted as ■ = EEEV and ▲ = RNV (data according to Burgueño et al., 2018). Places and number of bat sampling and alphavirus results are shown as ■ = EEEV and ▲ = RNV (this work).

In the present study, we re-analyzed 77 oral swabs collected in a previous study [32] from nine different bat species: *Desmodus rotundus*, *Eumops bonariensis*, *Molossops temminckii*, *Molossus*, *Molossus rufus*, *Tadarida brasiliensis*, *Eptesicus diminutus*, *Eptesicus furinalis,* and *Myotis* spp. Collections were carried out between 2013 and 2015 at five departments (Artigas, Maldonado, Montevideo, Rivera, and Rocha) of Uruguay (Figure 1, Table 1).

Species were identified upon capture using the last available national field guide, based on external characteristics [33]. Oral swabs were placed in cryotubes with 200 µL of viral buffer (AVL^®^ buffer from Qiagen, Hilden, Germany) and stored at room temperature before being taken to the laboratory, where they were processed immediately. After sampling, most bats were wing banded and released as part of a long-term study in the colony.

### 2.2. Total RNA Extraction and Generic nsP4 Amplification by Reverse Transcription and Nested Polymerase Chain Reaction (RT-Nested PCR)

Total viral nucleic acids of all oral swab samples were extracted using a commercial kit QIAmp^®^ Viral RNA (Qiagen, Hilden, Germany) according with manufacturer’s instructions. Five hundred microliters of the same AVL^®^ buffer was added to the cryotubes with the swab to complete a volume that was easy to handle. Additionally, 5.6 µL of RNA Carrier (Qiagen) was added and incubated 10 min at room temperature to continue with the manufacturer’s instructions. Nucleic acid extractions were aliquoted and frozen at −80 °C until the molecular analysis.

A 195 base pair fragment of the *nsP4* gene of alphaviruses was amplified by reverse transcription (RT), followed by nested polymerase chain reaction (nested PCR) assay, according to Sanchez-Seco et al. [34]. Reverse transcription was carried out as follows: 5 µL of total RNA was mixed with 40 pmol of primer Alpha 1−(5′-CKYTCYTCIGTRTGYTTIGTICCIGG-3′), 0.2 mM of each dNTP, and 4 µL of water and incubated at 65 °C/5 min, followed by 5 min on ice. An amount of 4 µL of 5× First Stand buffer, 1 µL of DTT (0.1 M), and 1 µL of SuperScript II ™ (Invitrogen, Waltham, MA, USA) was added to a final volume of 20 µL and incubated at 48 °C/60 min and 70 °C/15 min.

For first PCR round, 5 µL of cDNA was mixed with 40 pmol of each primer Alpha 1+ (5′-GAYGCITAYYTIGAYATGGTIGAIGG-3′) and Alpha 1−, 5 µL of 10× PCR buffer, 1.5 µL MgCl_2_ (50 mM), 0.2 mM of each dNTP, and 0.3 µL of Platinum^®^ Taq DNA Polymerase (Invitrogen, USA) to a final volume of 50 µL. Cycle conditions were one initial cycle of 94 °C/2 min, followed by 40 cycles of 94 °C/30 s, 52 °C/1 min and 72 °C/30 s. A final extension at 72 °C/5 min was performed.

The second PCR round was carried out in a final volume of 50 µL using 1 µL of the first-round product, mixed with 1.5 µL of MgCl_2_ (50 mM), 0.2 mM of each dNTPs, 5 µL of 10× PCR buffer, 0.3 µL of Platinum^®^ Taq DNA Polymerase (Invitrogen, Waltham, MA, USA), and 40 pmol of each primer: Alpha 2+ (5′-GIAAYTGYAAYGTIACICARATG-3′) and Alpha 2− (5′-GCRAAIARIGCIGCIGCYTYIGGICC-3′). Cycle conditions were the same as for the first round.

Two semi nested assays were performed with positive samples using a combination of primers from PCR round 1 and 2—Alpha 2+ (10 µM) with Alpha 1− (10 µM) and Alpha 1+ (10 µM) with Alpha 2− (10 µM)—to obtain a fragment of 303 bp and 372 bp, respectively. The volumes and cycles were under the same conditions as the nested PCR.

Total RNA extraction from an isolate of CHIKV was employed as a positive control. Negative controls were included in every assay. Visualization of the PCR products was performed by electrophoresis in a 1.5% agarose gel and stained with 1 µg/mL SybrSafe^®^ (Invitrogen, Carlsbad, CA, USA).

### 2.3. Sequencing and Phylogenetic Analysis

PCR products of alphavirus positive samples were purified and sequenced (plus and minus strands) using 5 pmol of the same primers used for amplification, at Macrogen Inc., Seoul, Korea. Sequences were reviewed and edited with BioEdit v7.2.5 software [35] and compared with GenBank database sequences using the nucleotide Basic Local Alignment Search Tool (BLASTn) (Table S1). *NsP4* sequence alignments were constructed using ClustalW [36]. Sequences of the positive samples and representative sequences of the alphavirus genus retrieved from GenBank were included to build the alignments. To estimate the most suitable model of nucleotide substitution, Modelgenerator v0.85 software was used. Phylogenetic reconstruction was conducted under the maximum likelihood (ML) criterion, using PhyML v3.0 software. Statistical supports of the tree nodes were calculated by the approximate likelihood ratio test (aLRT).

### 2.4. Cytochrome B Assay

Bat species confirmation of the alphavirus-positive individuals was accomplished by cytochrome b analysis on the same oral swab extracts used for virus detection. PCR amplification of a 400 base-pair of the cytochrome b gene was carried out according to Martins et al. [37]. Briefly, 2 µL of each extract was mixed with 50 pmol of primers Bat 14A (5′-TATTCCCTTTGCCGGTTTACAAGACC-3′) and Bat 17A (5′-AACCTCCTAGGAGACCCAGACAATT-3′), 5 µL of 10× PCR Buffer, 1.5 µL of MgCl_2_ (50 mM), 0.2 mM each dNTPs, and 0.2 µL of Platinum ™ Taq DNA Polymerase (Invitrogen, Waltham, MA, USA) to a final volume of 50 µL. Cycle conditions were one initial cycle of 94 °C/5 min, followed by 35 cycles of 94 °C/30 s, 48 °C for 45 s, 72 °C/1:10 min, and a final step of 72 °C/10 min. Visualization of the PCR products was performed as described above. Sanger sequencing was carried out with the same PCR primers, at Macrogen Inc., Seoul, Korea.

## 3. Results

Seventy-seven oral swab samples from nine autochthonous species of Uruguayan bats sampled in 2013 and 2015 were analyzed to detect alphavirus. From the 77 analyzed samples, 9 were positive to alphavirus. Positive samples corresponded to two bat species: *Tadarida brasiliensis* and *Myotis* spp. from Rivera and Artigas departments. All positive samples from Rivera department were obtained from the same roost (Usina Cuñapirú) within a three-day period in 2015 (Figure 1, Table 2 and Table S1).

**Figure 2:**
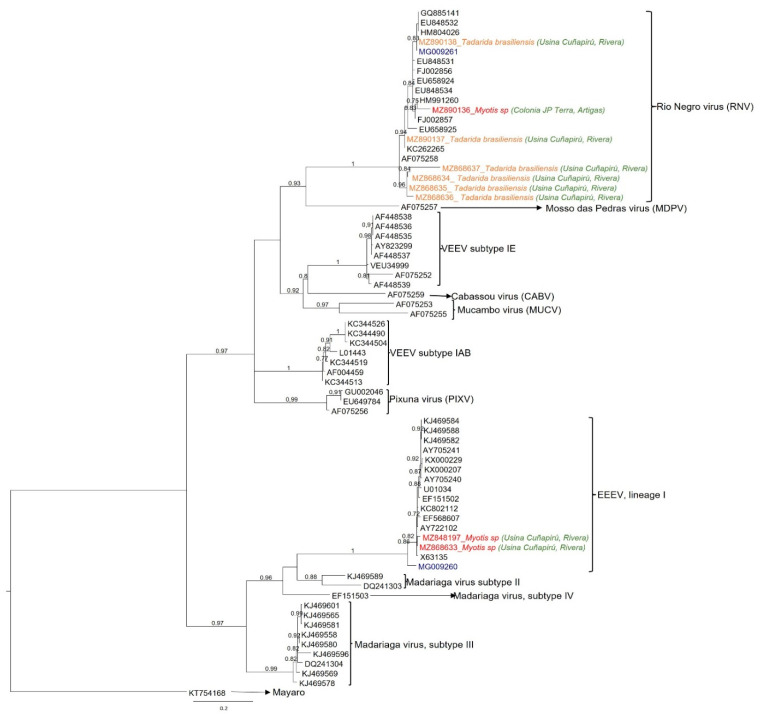
Maximum likelihood phylogenetic tree based on partial *nsP4* sequences. For comparison, sixty-two alphavirus sequences from different species were downloaded from GenBank, including the mosquito sequences previously reported in Uruguay (blue). Phylogeny was reconstructed under the GTR + Γ + I nucleotide substitution model. Mayaro virus was used as outgroup species. Clade supports were estimated through approximate likelihood ratio test (aLRT), and only those above 0.70 are shown. Accession numbers, bat species, and capture localities of the nine alphaviruses detected in this work are depicted in color: viruses found in *Tadarida brasiliensis*: orange; viruses found in *Myotis* spp.: red; capture sites: green; RNV and EEEV positive mosquitoes from Uruguay: blue.

Sequences from nested and/or semi-nested amplifications were compared with 62 alphavirus sequences of encephalitic alphaviruses from different antigenic complexes. The data set also included two sequences obtained in a previous study from mosquitoes in Uruguay (MG009261 and MG009260) [14] and other South American sequences from Argentina and Colombia. Maximum likelihood phylogeny (Figure 2) showed that seven viral sequences grouped with high statistical support (aLRT = 1) within the Rio Negro virus (RNV) clade, from the VEEV complex. The remaining two sequences clustered within lineage I eastern equine encephalitis virus, also with significant statistical support (aLRT = 1).

Out of the seven RNV-positive samples, six were detected in *T. brasiliensis* captured in 2015 at Rivera department and one from a *Myotis* spp. sampled at Colonia J.P. Terra (Artigas department) in 2013. Both EEEV-positive samples were from *Myotis* spp. also captured in 2015 at Rivera department (Figure 1 and Table 2).

Concerning the RNV clade, four sequences retrieved from *T. brasiliensis* (MZ868634, MZ868635, MZ868636, and MZ868637) form a monophyletic subclade (aLRT = 0.96). Meanwhile, two additional RNV (MZ890137 and MZ890138) obtained also from *T. brasiliensis* were grouped in a larger subclade (aLRT = 0.94), together with RNV sequences from rodents and mosquitoes captured in Argentina. This group also included sequence MZ890136 obtained from a *Myotis* spp. collected in Artigas, over 100 km away from Usina Cuñapirú and sampled two years before. In turn, the MZ890138 sequence, clustered together (aLTR = 0.83) with a RNV sequence (MG009261) obtained from *Culex pipiens* mosquitoes collected in 2014, at 350 km South (Canelones department) (Figure 1).

Among EEEV lineage I, the two sequences MZ848197 and MZ868633, from *Myotis* spp. captured in the same year (2015) and department (Rivera), grouped together with an aLRT support of 0.82. They clustered in a highly supported group (aLTR = 1) with a North American EEEV strain (X63135) and a sequence obtained from mosquitoes collected at Rio Negro department (approximately 250 km southwest from Usina de Cuñapirú, Figure 1). A previously reported EEEV from a mosquito pool collected in Colombia (KC802112) [38] also integrated the lineage of I clade, but it was not directly related to our bat sequences.

To confirm the bat species, a cytochrome B assay was carried out using the same oral swab extracts employed to detect alphaviruses. Enough extract was available from seven of nine swabs: four corresponded to *T. brasiliensis* (samples 26, 28, 38, and 39) and three to *Myotis* spp. (samples 11, 56, and 61) (Table 2). Sequences obtained were mostly of good quality; a Blastn search analysis showed that they agreed with the bat genus/species assigned by morphological taxonomy (Table S2). The only exception was 17A sequence from sample 39 (*T. brasiliensis*), which had low quality and produced a clearly unreliable result.

## 4. Discussion

Bats have long been indicated as potential reservoirs for arboviruses. Serologic evidence of arboviral infection, as well as virus detection or isolation in bats, has been reported in various studies from diverse geographical regions [19,39]. Regarding alphaviruses such as EEEV and VEEV, studies come mainly from North and Central America. Most of the evidence was found in frugivorous and insectivorous bats, but there are also reports in *Myotis* spp., *Eptesicus* sp. or *Desmodus rotundus,* which are autochthonous genus/species in our country (reviewed by Fagre 2019) [23,26].

Arbovirus studies in South American bats are scarce. Two recent reports from Colombia found genome and antigen of VEEV in frugivorous bats (*Artibeus planirostris* and *Sturrnira lillium*) [24,40]. By contrast, Bittar et al. analyzed 103 individuals and did not find evidence of flavivirus or alphavirus antibodies and/or genome in bats sampled from Bahia and Sao Paulo states (Brazil), but it should be noted that, regarding serology, the hemagglutination inhibition (HI) test has lower sensitivity than plaque reduction neutralization test (PRNT) for antibody assessment [41].

In the present study we report the genomic detection and identification of two different alphaviruses in oral swabs from bats captured in Northern Uruguay. In previous studies, our group identified RNV and EEEV in mosquitoes and horse serology, suggesting that these viruses may be circulating in enzootic cycles in our country [14]. The herein reported phylogenetic analysis identified RNV in two different bat species: *T. brasiliensis* (*n* = 6) and *Myotis* spp. (*n* = 1), captured in different localities (Figure 1 and Figure 2). This clade also included RNV from Argentina isolated from mosquitoes and rodents and from our previous finding in mosquitoes from southern Uruguay (Canelones department). Rio Negro virus sequences recovered from *T. brasiliensis* were not identical, despite having been collected from bats sharing the same shelter and sampled in a short timeframe. Only one sequence from *T. brasiliensis* was closely related with the one identified in mosquitoes captured 350 km apart and one year before. The other five sequences were less related, and four of them clustered in a monophyletic subclade. In turn, another RNV sequence was recovered from a *Myotis* spp. captured 100 km apart (Colona J.P. Terra, Artigas department), indicating that there could be several foci of viral activity in different bat populations. At the same time, we found RNV diversity in a single bat colony (Usina de Cuñapirú). Coincidently, most of the RNV positives come from Rivera department, where the Usina de Cuñapirú colony is located, and this is one of the departments with higher horse serology to alphavirus, according to a previous report from our group (Figure 1). Taken together, these results may indicate ongoing RNV activity in this area and deserve a more in-depth study involving simultaneous mosquito and bat trapping. Our previous seroprevalence study did not show alphavirus antibodies in horses from Artigas department, but we detected a RNV positive bat in Colonia J.P. Terra (Figure 1). This may be explained by a recent introduction of the virus or by a subsampling in our former study.

Two samples from *Myotis* spp. resulted as being positive to EEEV I (lineage I), both bats belonging to the Usina de Cuñapirú colony. These sequences appear closely related in the phylogeny and cluster in a well-supported clade with North American sequences and in a Uruguayan and a Colombian sequence, both retrieved from *Culex* spp. mosquitoes. Interestingly, EEEV- and RNV-positive bats were collected in the same shelter during the same field trip. This raises the possibility of two separate arboviral cycles coexisting in different bat species that share the same shelter. Even though lineage I EEEV is enzootic only on the east coast of North America, the Caribbean, and East Central America [42,43], our finding adds to recent reports in field-caught mosquitoes in Colombia and Uruguay [14,44]. It is possible that silent circulation or sporadic introductions of this viral lineage may have occurred. EEEV can cause serious epizootic or epidemic events; thus, the former detection in mosquitoes and the present finding in a vertebrate host is suggestive and is worth further investigation.

Field identification of bats was further confirmed through cytochrome B sequencing in most positive individuals. *Tadarida brasiliensis* was confirmed at the species level; however, *Myotis* spp. was only genus-level confirmed because *Myotis* taxonomy in the southern cone is still difficult, and genetic reference sequences are scarce. As an example, recently, a new *Myotis* species was described from individuals from Uruguay with no genetic material available [45].

Samples analyzed were not subjected to virus isolation, and we were not able to obtain complete genomes. Oral swab collections were carried out directly in AVL^®^ buffer, so virus isolation could not be achieved. In addition, samples were used to identify other viruses (herpesvirus, pneumovirus, and rhabdovirus) in a previous study [32]; thus, the remaining RNA was insufficient to attempt complete genome sequencing. This is a limitation in our study; however, we could accurately identify the viruses and obtain a reliable phylogeny, showing two alphavirus species infecting Uruguayan bats.

Evidence supporting bats as true arboviral amplifying hosts is still scarce and remains difficult to collect. To consider a particular vertebrate as an arbovirus reservoir host, several criteria should be met. These include the periodic isolation of the virus from the vertebrate in absence of seasonal vector activity and the coincidence of transmission with vector activity. Additionally, the vertebrate host must develop enough viremia (quantitatively and in time-extension) for the hematophagous arthropod to acquire an infectious bloodmeal, thus leading to virus transmission [46]. Our findings reveal that bats can be infected by the EEEV and RNV alphaviruses and that chiropterans could participate in their natural cycle, as viral amplifiers or as dead-end hosts. To address the role of these mammals in the viral cycles, additional studies are ongoing, including viral detection and blood meal analysis in mosquitoes collected in the vicinity of the colonies, together with viremia and serology in bats.

## Figures and Tables

**Table 1:** Georeferenced locality, year, and number of bat families and species sampled.

Family	Genus and/or Species	# of Samples	Department	Georeference	Year
Molossidae	*Molossus rufus*	1	Artigas	30.23 S, 57.57 W	2013–2015
	*Molossus molossus*	1			
	*Eumops bonariensis*	1			
	*Molossops temminckii*	2			
Vespertilionidae	*Myotis* spp.	2			
	*Eptesicus furinalis*	2			
	*Eptesicus diminutus*	2			
Phyllostomidae	*Desmodus rotundus*	19	Maldonado	34.48 S, 54.61 W	2013
Vespertilionidae	*Eptesicus furinalis*	1	Montevideo	34.86 S, 56.20 W	2015
Molossidae	*Tadarida brasiliensis*	28	Rivera	31.52 S, 55.59 W	2015
Vespertilionidae	*Myotis* spp.	13			
Molossidae	*Molossus molossus*	3	Rocha	34.29 S, 54.06 W	2013
Vespertilionidae	*Eptesicus furinalis*	2			

**Table 2:** Alphavirus results. Bat families and species, GenBank accession numbers for sequences, site, and year of collection.

Family	Genus/Species	Result	Sample (Lab. N°)	GB Accession Number	Department	Collection Year
Molossidae	*Tadarida brasiliensis*	RNV	26	MZ868634	Rivera	2015
	*Tadarida brasiliensis*	RNV	28	MZ868635	Rivera	2015
	*Tadarida brasiliensis*	RNV	31	MZ868637	Rivera	2015
	*Tadarida brasiliensis*	RNV	35	MZ890137	Rivera	2015
	*Tadarida brasiliensis*	RNV	38	MZ868636	Rivera	2015
	*Tadarida brasiliensis*	RNV	39	MZ890138	Rivera	2015
Vespertilionidae	*Myotis* spp.	RNV	11	MZ890136	Artigas	2013
	*Myotis* spp.	EEEV	56	MZ848197	Rivera	2015
	*Myotis* spp.	EEEV	61	MZ868633	Rivera	2015

## Data Availability

Sequences reported in this study were submitted to GenBank with accession numbers: MZ868634, MZ868635, MZ868637, MZ890137, MZ868636, MZ890138, MZ890136, MZ848197, MZ868633, OM038093, OM038092, M038090, OM038089, OM038091, OM001113, OM001112.

## Supplementary Materials

The following supporting information can be downloaded at: https://www.mdpi.com/article/10.3390/v14020269/s1, Table S1, Blastn analysis of sequences from alphavirus-positive oral swabs. Table S2. Cytochrome B analysis carried on the alphavirus positive oral swabs.
