# Supplementary material for: Alphavirus Identification in Neotropical Bats
Source: Viruses. 2022 Jan 28;14(2):269. doi: 10.3390/v14020269 (PMC8877408; doi:10.3390/v14020269)
Supplement: Supplementary file 1 [file viruses-14-00269-s001.zip › viruses-1555296-supplementary.pdf]

Supplementary Table S1: Blastn analysis of sequences from alphavirus-positive oral swabs. Morphometric identification, location and collection date are shown. Blastn analysis was done with the default settings. Percentage identity and accession number of the most similar sequence are indicated.

Sample (lab. number)	Genus/species	Location	Date	GenBank Acc. number	Blastn (nucleotide)	Per. identity (%)	First hit accession number
26	*Tadarida brasiliensis*	Usina Cuñapirú	2015	MZ868634	RNV	97.05	NC_038674.1
28	*Tadarida brasiliensis*	Usina Cuñapirú	2015	MZ868635	RNV	98.82	NC_038674.1
31	*Tadarida brasiliensis*	Usina Cuñapirú	2015	MZ868637	RNV	93.83	NC_038674.1
35	*Tadarida brasiliensis*	Usina Cuñapirú	2015	MZ890137	RNV	100	MG009261.1
38	*Tadarida brasiliensis*	Usina Cuñapirú	2015	MZ868636	RNV	97.68	NC_038674.1
39	*Tadarida brasiliensis*	Usina Cuñapirú	2015	MZ890138	RNV	100	MG009261.1
11	*Myotis sp*	Colonia J.P.Terra	2013	MZ890136	RNV	95.27	MG009261.1
56	*Myotis sp*	Usina Cuñapirú	2015	MZ848197	EEEV	99.08	KJ469595.1
61	*Myotis sp*	Usina Cuñapirú	2015	MZ868633	EEEV	99.77	KJ469595.1

Supplementary Table S2: Cytochrome B analysis carried on the alphavirus positive oral swabs. Location, collection date and morphometric identification are shown. Blastn analysis was done with the default settings. Percentage identity and accession number of the most similar sequence are indicated.

Sample (lab. number)	Genus/species (by morphology)	Location	Date	GenBank Acc. number	CytB - Blastn (nucleotide)	Per. identity (%)	First hit accession number
11	*Myotis sp*	Colonia J.P.Terra	2013	OM038093	*Myotis sp* *(levis, albescens, nigricans)*	94.39	JX130520
26	*Tadarida brasiliensis*	Usina Cuñapirú	2015	OM038092	*Tadarida brasiliensis*	86.36	MF135762
28	*Tadarida brasiliensis*	Usina Cuñapirú	2015	OM038090	*Tadarida brasiliensis*	84.81	KP134553
38	*Tadarida brasiliensis*	Usina Cuñapirú	2015	OM038089	*Tadarida brasiliensis*	78.11	KP134553
39	*Tadarida brasiliensis*	Usina Cuñapirú	2015	OM038091	*Tadarida brasiliensis*	77.13	MG029505
56	*Myotis sp*	Usina Cuñapirú	2015	OM001113	*Myotis sp* *(levis, nigricans)*	97.70	JX130475
61	*Myotis sp*	Usina Cuñapirú	2015	OM001112	*Myotis sp* *(levis, nigricans)*	99.23	MT262871

Sample (lab. number)	Genus/species (by morphology)	Location	Date	GenBank Acc. number	CytB - Blastn (nucleotide)	Per. identity (%)	First hit accession number
11	*Myotis sp*	Colonia J.P.Terra	2013	OM038093	*Myotis sp* *(levis, albescens, nigricans)*	94.39	JX130520
26	*Tadarida brasiliensis*	Usina Cuñapirú	2015	OM038092	*Tadarida brasiliensis*	86.36	MF135762
28	*Tadarida brasiliensis*	Usina Cuñapirú	2015	OM038090	*Tadarida brasiliensis*	84.81	KP134553
38	*Tadarida brasiliensis*	Usina Cuñapirú	2015	OM038089	*Tadarida brasiliensis*	78.11	KP134553
39	*Tadarida brasiliensis*	Usina Cuñapirú	2015	OM038091	*Tadarida brasiliensis*	77.13	MG029505
56	*Myotis sp*	Usina Cuñapirú	2015	OM001113	*Myotis sp* *(levis, nigricans)*	97.70	JX130475
61	*Myotis sp*	Usina Cuñapirú	2015	OM001112	*Myotis sp* *(levis, nigricans)*	99.23	MT262871
